# Biogenic Amines, Phenolic, and Aroma-Related Compounds of Unroasted and Roasted Cocoa Beans with Different Origin

**DOI:** 10.3390/foods8080306

**Published:** 2019-08-01

**Authors:** Umile Gianfranco Spizzirri, Francesca Ieri, Margherita Campo, Donatella Paolino, Donatella Restuccia, Annalisa Romani

**Affiliations:** 1 Department of Pharmacy, Health and Nutritional Sciences, University of Calabria, I-87036 Rende (CS), Italy; 2 Department of Statistic, Informatics and Applications “G. Parenti” (DiSIA)—University of Florence, Phytolab Laboratory, via Ugo Schiff 6, 50019 Sesto Fiorentino (FI), Italy; 3 Department of Experimental and Clinical Medicine, University of Catanzaro “Magna Græcia”, 88100 Catanzaro, Italy

**Keywords:** cocoa nibs, roasting, bioactive amines, polyphenols, volatile organic compounds, geographical areas

## Abstract

Biogenic amines (BAs), polyphenols, and aroma compounds were determined by chromatographic techniques in cocoa beans of different geographical origin, also considering the effect of roasting (95, 110, and 125 °C). In all samples, methylxantines (2.22–12.3 mg kg^−1^) were the most abundant followed by procyanidins (0.69–9.39 mg kg^−1^) and epicatechin (0.16–3.12 mg kg^−1^), all reduced by heat treatments. Volatile organic compounds and BAs showed variable levels and distributions. Although showing the highest BAs total content (28.8 mg kg^−1^), Criollo variety presented a good aroma profile, suggesting a possible processing without roasting. Heat treatments influenced the aroma compounds especially for Nicaragua sample, increasing more than two-fold desirable aldehydes and pyrazines formed during the Maillard cascade and the Strecker degradation. As the temperature increased, the concentration of BAs already present in raw samples increased as well, although never reaching hazardous levels.

## 1. Introduction

Cocoa beans represent the basic raw material in the production of chocolate and cocoa-based products. During processing, cocoa beans undergo important manipulations, including fermentation and roasting, which drastically influence the quality of the final product. During fermentation, cocoa beans are exposed to the action of various microorganisms and enzymes, while in the roasting process, high temperatures determine important modifications on the cocoa bean’s composition [1].

Cocoa is a food rich in polyphenols, mainly flavonoids, procyanidins, and flavan-3-ols. The preservation or enhancement of cocoa procyanidins is of great importance since these compounds, despite their poor bioavailability, have been related to the health beneficial effects of cocoa, particularly in cardiovascular diseases [2]. Polyphenol and xanthine content in cocoa seeds changes during ripening and during the processing phases [3]. Microbial activity during fermentation and the drying process contribute to settling the amounts of theobromine and caffeine and their relative abundances, polyphenol amounts, in particular of catechin and epicatechin, and the amounts of organic acids, sugars, mannitol, ethanol, and alkaloids, thus influencing the quality and the biological properties of the finished product [4]. At the beginning of fermentation, during the first three days, the highest contents of total phenolic compounds and total anthocyanins prevailed in cocoa beans. Finally, at the end of cocoa beans fermentation, the lowest contents of total phenolic compounds, and total anthocyanins, were observed [5].

In addition, roasting temperature has been seen to have effects on the flavanols amounts causing losses and structural modifications, particularly epimerization of both monomers and polymers. At high roasting temperatures, a progressive loss of (−)-epicatechin and (+)-catechin and an increase in (−)-catechin were observed as a result of heat-related epimerization from (−)-epicatechin; additionally, a temperature-related epimerization of procyanidin dimers has been reported [6,7]. These structural modifications could have negative effects on the biological properties of the product, being (−)-epicatechin the most bioavailable isomer and (−)-catechin the one with the lowest bioavailability. Roasting processes may also cause reduction of the content of hydroxycynnamic compounds, clovamide in particular [8], with a possible further reduction of the antioxidant activity.

The secret of the flavor of chocolate, so highly appreciated worldwide, resides mainly in its volatile aromatic fraction. Its complex composition depends on the cocoa bean genotype and is the consequence of several processes [9,10]. To date, descriptive studies have identified >600 volatile compounds in cocoa and chocolate products [11,12], mainly pyrazines, esters, amines and amides, acids, and hydrocarbons. Besides cocoa flavor precursors, also toxic molecules, such as biogenic amines (BAs), can be produced during processing. BAs are a class of organic, basic, nitrogenous compounds with low molecular weight, are usually part of bioactive molecules of cocoa beans and derivatives. They mainly derived by decarboxylation of corresponding amino acids, due to the action of suitable enzymes widely distributed in spoilage bacteria and other microorganisms, as well as in naturally occurring and/or artificially added bacteria involved in food fermentation [5,13]. Although several amines (i.e., natural polyamines) are present in living cells and contribute to promoting many human physiological functions, these compounds can represent a serious health hazard for humans, when present in food in significant amounts or ingested in the presence of potentiating factors, such as amine oxidase-inhibiting drugs, alcohol, and gastrointestinal diseases. Then, their attendance in foodstuffs is often undesirable, because often associated with several of pathological syndromes, such as headaches, respiratory distress, heart palpitations, hypo- or hypertension and several allergenic disorders [14]. In particular, tyramine, β-phenylethylamine, and histamine have been considered as the initiators of hypertension and dietary-induced migraines, while the neurotransmitter serotonin is essential in the regulation of appetite, body temperature, and sleep [15].

Generally speaking, a variety of factors determining cocoa and cocoa derivatives quality are strongly related to the cocoa beans processing, from the opening of the fruit until the end of industrial processes [16]. In addition, qualitative characteristics of the cocoa beans are a consequence of the differences in the farming practices regarding growing, fermenting, and drying the cocoa beans, with significant differences sometimes found in samples from the same country [16,17].

Recently, it has been reported that in cocoa beans the amino acid oxidative decarboxylation can be also obtained during food processing suggesting a new chemical, heat-induced formation of BAs [18]. It follows that, in addition to the amino acid catabolism produced by microorganisms, amino acids can also be degraded chemically as a consequence of thermal treatment of foods [19]. These reactions are responsible for the formation of taste and flavor compounds, reducing, at the same time, the concentration of essential amino acids and contributing to the accumulation of compounds that may be dangerous for consumers, such as BAs [20]. It follows that two main reasons can be underlined accounting for the analysis of BAs in foods: first their potential toxicity; second the possibility of using them as food quality markers as their concentration can be related with the hygienic-sanitary quality of the process and with the freshness of the raw materials and the processed products. As BAs have been widely exploited as important indicators of safety and quality in a variety of foods, many papers appeared in recent years reporting their quantitative determination in many fermented and non-fermented foods, including cocoa and its derivatives [13].

In this work, the quantitative determination of biogenic amines, xanthine, and polyphenol molecules was performed by liquid chromatography (LC) techniques on cocoa beans from different origin. The aroma profile of cocoa nibs was investigated by headspace solid-phase micro-extraction (HS-SPME) combined with gas chromatography-mass spectrometry (GC-MS). Additionally, the same compounds were monitored after roasting process at different temperatures to establish a possible correlation between heating and different biomolecules profiles.

## 2. Materials and Methods

### 2.1. Samples

Seven cocoa beans samples from different world areas and years were considered as reported in Table 1. All fruits studied in this work were considered as well fermented. Approximately 500 g portions of Coviriali and O’Payo cocoa beans of uniform size were random selected from 50 kg of each nib variety peeled and roasted in a forced airflow-drying oven ROASTER CENTOVENTI vertiflow^®^ system (Selmi Chocoloate Machinery, Cuneo, Italy) at Meraviglie S.r.l. (Verona, Italy). Applied in these studies, heat treatment parameters were chosen to obtain a range of roasted beans with acceptable physico-chemical and sensory properties. The parameters of thermal processing were optimized to avoid over-roasting, varying time of roasting as soon as bean cracking occurs, as follows: temperatures of 95, 110, and 125 °C for 60, 30, and 20 min respectively. Samples from all bean types were prepared using differentiated methods according to the analyses carried out as reported in the subsections of the experimental section.

### 2.2. Chemicals

BAs spermine (SPM, tetrahydrochloride), spermidine (SPD, trihydrochloride), putrescine (PUT, dihydrochloride), histamine (HIM, dihydrochloride), tyramine (TYR, hydrochloride), β-phenylethylamine (PHE, hydrochloride), cadaverine (CAD, hydrochloride), as well as dansyl chloride, ammonia (30%), perchloric acid, and LC solvents (acetonitrile and methanol LC grade) were purchased from Sigma-Aldrich (Milford, MA, USA). Ultrapure water was obtained from Milli-Q System (Millipore Corp., Milford, MA, USA). Filters (0.45 and 0.20 μm) were purchased from Sigma-Aldrich. SPE C18 cartridges (0.5 g) were obtained from Supelco Inc. (Bellefonte, PA, USA). All GC chemicals were from Sigma-Aldrich (Milford, MA, USA). The HPLC grade standards (±)-catechin hydrate, theobromine, caffeic acid, quercetin-3-glucoside were purchased from Sigma-Aldrich (Milford, MA, USA).

### 2.3. Samples Preparation for the Analysis of Polyphenols and Xanthines

The peeled cocoa beans were crushed in a mortar, then 1.0 g accurately weighed of crushed material was extracted in 10.0 mL of a solution EtOH:H_2_O 70:30 at pH 3.2 by addition of HCOOH, at room temperature, for 24 h under stirring. The solid material was removed by filtration under vacuum and the extracts analyzed by high performance liquid chromatography coupled with diode array detection and electrospray ionization mass spectrometer (HPLC-DAD-ESI-MS) and by high performance liquid chromatography coupled with diode array detection and fluorescence detector (HPLC-DAD-FLD).

### 2.4. Chromatographic Conditions Xanthine and Polyphenol Determination

The method used for the quali-quantitative analysis, and described below, was optimized according to literature data and previous studies of this research group about polyphenolic compounds and xanthines, and modifying them based on the specific results [8,21,22,23]. The analyses were performed using a HP-1200 Liquid Chromatograph with a DAD and a fluorescence detector and a HP-1100 MSD API Electrospray (Agilent Technologies, Palo Alto, CA, USA) operating in negative and positive ionization mode. Gas temperature was 350 °C, flow rate 10.0 L/min, nebulizer pressure 30 psi, quadrupole temperature 300 °C, capillary voltage 3500 V, and fragmentor 120 eV.

**Figure 1:**
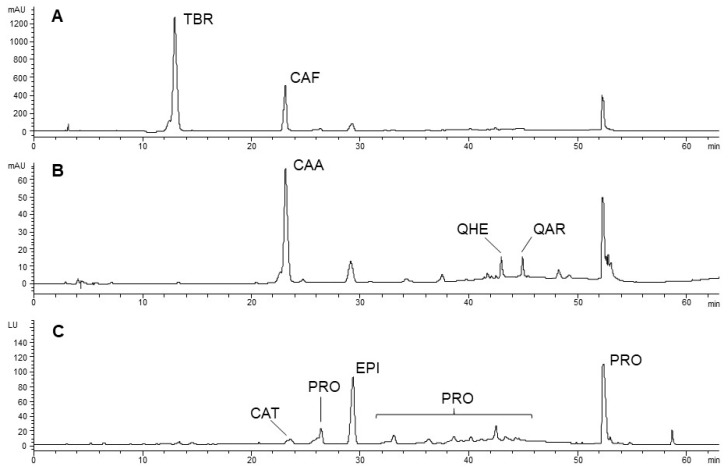
Chromatographic profile of raw O’Payo bean extract (2016): diode array detection (DAD) 280 nm (**A**) and 330 nm (**B**); fluorescence detector (FLD) ex. 280 nm; em. 315 nm (**C**). TBR—Theobromine; CAF—Caffeine; PRO—Procyanidins; CAA—Caffeoyl aspartic acid; QHE—Quercetin hexoside; QAR—Quercetin arabinoside; CAT—Catechin; EPI—Epicatechin.

For the chromatographic separation a Luna C18 250 × 4.60 mm, 5 µm (Phenomenex, Torrance, CA, USA) column was used operating at 26 °C. A multistep linear solvent gradient starting from 95% H_2_O at pH 3.2 by addition of HCOOH (A), up to 100% CH_3_CN (B) was performed with a flow rate of 0.8 mL min^−1^ over a 63 min period. In detail, the applied gradient started with 95% A; from 95% A to 85% A in 20 min; isocratic elution 85% A until 30 min; from 85% A to 75% A in 9 min; isocratic elution 75% A until 47 min; from 75% A to 15% A in 2 min; isocratic elution 15% A until 53 min; from 15% A to 0% A in 2 min; 0% A until 60 min; from 0% A to the initial conditions in 3 min. Figure 1 reports on the chromatographic profile of raw O’Payo bean extract (2016) by DAD at 280 nm (A) and 330 nm (B) and FLD ex. 280 nm; em. 315 nm (C).

### 2.5. Calibration

Quantitation of xanthines, flavonols, hydroxycinnamic derivatives, and procyaninides was performed by HPLC-DAD using five-point regression curves built with the available standards. Curves with an r^2^ > 0.9998 were considered. Calibration was performed at the wavelength of the maximum UV-Vis absorbance, applying the correction of molecular weight. In particular, the extinction coefficient of each quantified compound being comparable to that of the specific standard used for its calibration, the weights in mg were calculated by multiplying the weight obtained from the calibration by a correction factor given by the ratio between the molecular weight of the compound and the molecular weight of the standard used for its calibration. In particular, xanthines were calibrated at 280 nm using theobromine as reference; procyanidins were calibrated at 280 nm using catechin hydrate as reference; hydroxycinnamic derivatives were calibrated at 330 nm using caffeic acid as reference; flavanols were calibrated at 350 nm using quercetin as reference. The quantitation of catechin and epicatechin was performed by HPLC-FLD, using a five point calibration curve (r^2^ = 0.9999) built with standard solutions of catechin hydrate. The fluorescence detector was set as follows: excitation wavelength 280 nm; emission wavelength 315 nm [24].

### 2.6. HS-SPME-GC-MS Analyses

Headspace solid-phase micro-extraction combined with gas chromatography–mass spectrometry (HS-SPME-GC-MS) was selected as the most suitable technique to recover and analyze the Volatile Organic Compounds (VOCs) in peeled cocoa beans samples. Samples were ground and homogenous powders were obtained. A total of 1 g of the powdered sample, was placed into a 20-mL screw cap vial fitted with PTFE/silicone septa. An Internal Standard (IS) in suitable amount was added to each sample (IS: ethylacetate-D8; 1-Butanol-D10; ethyl hexanoate-D11; 5-methyl-hexanol; acetic acid-D3; Hexanoic acid-D11; 3,4-Dimethylphenol;). The Internal Standard was used for normalizing the analyte responses over the area of the IS, to minimize the instrumental error during the time of analysis.

After some trials aimed at optimizing amounts of sample, exposure time, and temperature, SPME conditions were set as follows: after 5 min of equilibration at 60 °C, VOCs were absorbed exposing a 1-cm divinilbenzene/carboxen/polydimethylsiloxane SPME fiber (DVB/CAR/PDMS by Supelco) for 15 min into the vial headspace under orbital shaking (500 rpm) and then immediately desorbed at 280 °C in a gas chromatograph injection port operating in split less mode. The chromatographic analysis was performed in a GC system coupled to quadrupole mass spectrometry using an Agilent 7890a GC equipped with a 5975C MSD (Agilent Technologies, Palo Alto, CA, USA). The separation of analytes was achieved by an Agilent DB InnoWAX column (length 50 m, id 0.20 μm, df 0.40 μm). Chromatographic conditions were: initial temperature 40 °C, then 10 °C min^−1^ up to 260 °C, hold for 6.6 min. Compounds were tentatively identified by comparing calculated Kovats retention index and mass spectra of each peak with those reported in mass spectral databases, namely the standard NIST08/Wiley98 libraries. Quadrupole MS operated in full-scan mode from which the specific ions of the analyte were extracted. Only the compounds with higher intensity were identified in order to select major compounds over a complex mixture of VOCs. Each sample was analyzed in triplicate.

### 2.7. Amine Standard Solutions and Calibration

**Figure 2:**
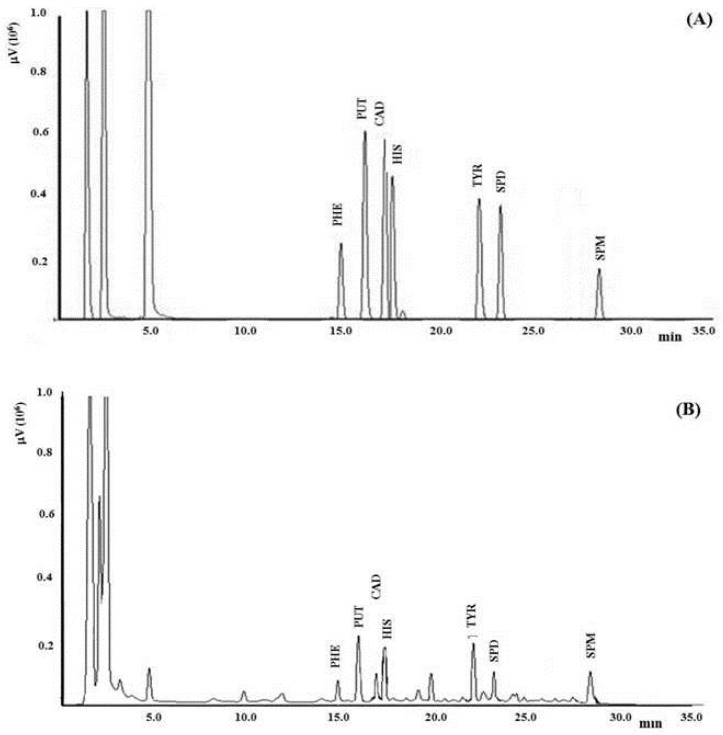
LC-UV chromatogram of biogenic amines (BAs) standard mixture at concentration of 100 µg mL^−1^ (**A**) and sample 1 (**B**). The chromatogram was obtained employing gradient conditions as specified in the Materials and Methods section.

A calibration curve was built starting from 1.0 mg mL^−1^ standard solution of each amine in purified water and preparing 12 BAs standard mixtures to a final volume of 25 mL employing HClO_4_ 0.6 mol L^−1^. Amine final concentrations were 0.1, 0.5, 0.8, 2.0, 4.0, 5.0, 10.0, 16.0, 25.0, 50.0, 75.0, and 100.0 μg mL^−1^. The comparison between the retention times of peaks of samples and standard solutions allowed the identification of each BA. Standard concentration against peak area allowed to build a calibration plot, and six independent replicates for each concentration level were performed. Moreover, the matrix effect was evaluated by comparison of external calibration plots, depicting concentration of standard solutions versus peak area, with standard addition method plots, depicting peak area versus concentration of standard solutions added to the sample. No significant matrix effect was recorded because of the slopes of the two plots were not significantly different. Quantitative determination was then accomplished by direct interpolation in the external calibration plot of each BA. Chromatogram of a standard mixture of BAs is displayed in Figure 2A.

### 2.8. BAs Extraction and Purification

The extraction of BAs from peeled cocoa beans samples was performed by adding 20 mL of HClO_4_ 0.6 mol L^−1^ to about 5.0 g of grounded sample, in a 50.0 mL test tube. The mixture was homogenized (vortex at 40 Hz for 40 min), centrifuged (10,000× *g* for 20 min), filtered (syringe filter 0.20 µm), collected in a plastic vial and purified by SPE on a C_18_ sorbent (conditioning: 2.0 mL of H_2_O and 2.0 mL (two times) of CH_3_OH; loading: 5.0 mL of the basified sample; washing: 2.0 mL of NH_4_OH at pH 11.0; eluting: 2.0 mL (two times) of CH_3_OH). Nitrogen gas was employed to dry eluting solution providing a solid residue that was re-dissolved in a plastic test tube with 1.3 mL of extraction solvent.

To perform recovery experiments sample 1 was spiked, before the extraction procedure, with an aliquot of standard mixture of BAs. Specifically, 5.0 g of peeled cocoa beans were spiked with 1.0 mL of 25.0 mg L^−1^ BAs standard solution. Method validation was obtained in terms of recovery percentages, linearity, intra- and inter-day repeatability, limits of quantification and limits of detection (LOQs and LODs), to confirm analytical suitability [25].

Dansylation reaction was performed by adding at 1.0 mL of standard solution (or acid sample extract spiked with BAs or acid sample extract) 200 µL of NaOH 2.0 mol L^−1^, 300 µL of saturated NaHCO_3_ solution and 2.0 mL of dansyl-chloride solution (10.0 mg mL^−1^ in acetone prepared just before use). After 30 min, dansyl-chloride in excess was removed with 100 µL of NH_4_OH 25% (v/v) and the suspension filtered by a 0.45 µm syringe filters. Finally, 20 µL was injected for LC-UV analysis. Figure 2B shows a chromatogram of sample 1.

### 2.9. Chromatographic Conditions for BAs Quantification

Jasco PU-2080 instrument equipped with a Rheodyne 7725 injector with a 20 mL sample loop and a gradient pump (PU-2089 plus, Jasco Inc., Easton, MD, USA) was employed to obtain the chromatograms. The system was interfaced with an UV detector operating at *λ* = 254 nm (UV-2075, Jasco Inc., Easton, MD, USA). Data were collected and analyzed with an integrator Jasco-Borwin1. A reverse-phase C18 column (250 mm × 4.6 I.D., 5 mm) (Supelco Inc., Bellefonte, PA, USA) equipped with a C18 guard-pak (10 mm × 4.6 I.D., 5 mm) were used (Supelco Inc., Bellefonte, PA, USA) for separation of BAs. Two solvent reservoirs containing (A) purified water and (B) acetonitrile were used to separate all the BAs with a gradient elution which began with 3 min of isocratic program A-B 50:50 (v/v) reaching after 20 min A-B 10:90 (v/v). Then 3 min of isocratic elution was carried out and 4 min further where necessary to restore again the starting conditions (A-B 50:50, v/v). A constant flow at 1.2 mL min^−1^ was employed.

### 2.10. Statistical Analyses

All analyses were performed in triplicate and data were expressed as mean ± relative standard deviations (RSD). Studies of the correlation coefficient and linear regression, calculation of average, assessment of repeatability, standard deviation, and RSD were performed using Microsoft Excel 2010 software. Significance was performed using a one-way analysis of variance (ANOVA) test, employing Duncan’s multiple range test at significance level *p* < 0.05.

## 3. Results and Discussion

### 3.1. Polyphenol Content in Cocoa Beans

To identify the phenolic compounds and xanthines, UV-Vis absorption spectra, mass spectra and literature data were used and combined for tentative identification of the analytes. In the present study, catechin and epicatechin were quantified through calibration by using a FLD detector, whereas oligomeric and polymeric procyanidins were quantified through DAD calibration. FLD calibration was used for catechin and epicatechin because of its higher sensibility and specificity, needed for catechin in particular, that often partially coelutes with caffeine, and is always present in low amounts with respect to this latter (average amount of catechin with respect to caffeine: 5.2% in the analyzed samples). In these conditions, the fact that both catechin and caffeine have also very similar wavelengths of maximum UV-Vis absorption hinders a correct quantification of the flavanol by using a DAD detector. Conversely, unlike caffeine, catechin and epicatechin emit a very intense fluorescence signal in the experimental conditions (excitation wavelength 280 nm; emission wavelength 315 nm), easily detectable and measurable also in presence of methylxanthines [24,26]. On the other hand, for the calibration of the other procyanidins a DAD detector is needed, because fluorescence detection is insensitive to procyanidins containing a gallic acid ester and/or gallocatechins as monomeric units [23]. In this case, the use of a fluorescence detector could lead to an underestimation of the total procyanidin content.

In Table 2 polyphenol distributions and total amounts in fermented, not-roasted cocoa beans of different origin are reported. Xanthines, theobromine in particular, are the most abundant compounds and their amounts decrease with increasing roasting temperature, after an initial slight increase by roasting at 95 °C, probably due to a further loss of water after the drying process.

Among the raw samples analyzed, the highest in polyphenols were 1^nr^ (Camino verde 2015), 3^nr^ (Coviriali 2015) and 6^nr^ (O’Payo 2016), respectively with 10.67, 10.53, and 12.96 mg g^−1^ total polyphenols. In all samples, it is possible to observe a clear predominance of epicatechin with respect to catechin; the highest [epicatechin]:[catechin] ratio (27.9) was found for the raw sample from Madagascar (2015), but its low content in polyphenols (1.44 mg g^−1^) suggests a lower quality with respect to the other samples under study. The other two samples harvested in 2015 (Camino verde and Coviriali) appear not to contain catechin, so it was impossible to evaluate the ratio even though the epicatechin amount appears to be high in particular for the Camino verde sample (2.84 mg g^−1^). The lowest [epicatechin]/[catechin] ratio was found for the raw samples Cheni and Criollo (5.0 for both the samples).

For Coviriali 2016 and O’Payo 2016 beans it was possible to compare the polyphenols contents after roasting at 110 °C, confirming that the total amounts of polyphenols significantly decrease with respect to the raw samples; Coviriali beans were roasted also at a higher temperature (125 °C) with a further decreasing of total polyphenols. According to previously reported data [27,28], also [epicatechin]/[catechin] ratios follow the same trend depending on the roasting process at high temperature. Procyanidins, quantified as catechin equivalents, are the most abundant polyphenols in all samples and their amounts also appear to be negatively influenced by the roasting process [28]. Two flavonolic compounds were found, quercetin hexoside and quercetin arabinoside, in low amounts and apparently not depending on the roasting temperature. The only hydroxycynnamic derivative present in the extracts was caffeoyl aspartic acid [3].

The characteristics of cocoa beans and derived food products, such as hydrophilic and volatile secondary metabolites profile, organoleptic properties, antioxidant activity etc., depend not only on fermentation and processing methods, but also on several variables related to genetics, geographical regions of cultivation, agronomical practices, and climatic conditions [29,30]. In particular, concerning phenolic compounds, the low amount of total polyphenols (1.28 mg g^−1^) found for Criollo variety is reliable based on literature data that identify this variety as the one with the lowest content of polyphenols [31,32]. The Forastero variety includes also Nacional Forastero that is the Forastero variety cultivated in northern Ecuador [31]. The analyzed Forastero samples were harvested in 2015 (Nacional Forastero from Ecuador, sample 1^nr^, and Forastero from Junìn region, Peru, sample 3^nr^) and 2016 (Forastero from Junìn region, sample 4^nr^, and Forastero from Satipo region, Peru, sample 5^nr^). For Forastero beans harvested in 2015, no significant difference was found between their contents of total polyphenols; interestingly, catechin was not detected in either of the two samples. Conversely, statistically significant differences were found between epicatechin and procyanidins contents. For Forastero beans harvested in 2016, total polyphenols are higher in the sample from Satipo region than in the one from Junìn region, but it must be taken into consideration that the total polyphenols content varies also according to climate variations, thus possibly from one year to another, as it can be seen for the two Junìn region samples of 2015 and 2016 (10.55 and 5.16 mg/g total polyphenols respectively). Moreover, a small difference was found between their contents of catechin, whereas the difference between epicatechin amounts is more evident. The differences between polyphenolic compositions are evident for the two samples of Trinitario variety from Sambirano region, Madagascar (sample 2^nr^) and Bali, Indonesia (sample 6^nr^). Sample 6^nr^, not roasted, is the highest in polyphenols among all the samples analyzed in the present study (12.96 mg/g total polyphenols), and its polyphenols content consists mainly of procyanidins (9.17 mg/g). Sample 2^nr^ shows a consistently lower total polyphenols content (1.44 mg/g), of which 1.00 mg/g procyanidins remaining the most represented polyphenolic subclass. Monomeric flavanols are also higher in the sample from Indonesia, but the epicatechin/catechin ratio is better for the other sample (12.9 vs. 27.9). Again, it must be noted that the samples 2^nr^ and 6^nr^ differ not only concerning their geographical origins but also regarding the years of production (2015 and 2016).

### 3.2. VOCs in Cocoa Beans

After a successful fermentation process, it is necessary to reduce the water content of the cocoa seeds to between 5% and 8% and this is achieved by drying [33]. The drying process is not only important in preserving the cocoa seeds but also plays a very crucial role in the development of cocoa flavor and the overall quality of the raw cocoa seeds. Cocoa is dried to minimize the formation of molds and to reduce the acid level and astringency of the beans. Then the roasting of the cocoa seeds takes place in the consumer countries. Among the cacao beans analyzed, the 7^nr^ sample (Criollo) beans will be used by a chocolate maker that produces raw chocolate, so beans will be not roasted and never reach temperatures of more than 42 °C. Especially for this product, the quality of cocoa beans is a very big determinant of the final taste of the chocolate. Cocoa flavor resides in volatile fraction, which is composed of a complex mixture of up to 600 compounds with new research continuously increasing this number [12].

HS-SPME coupled to GC-MS has proven a valuable tool for analysis of volatile and semi-volatile compounds from cocoa and chocolate products. The technique is very sensitive to experimental conditions and in this study, the DVB/CAR-PDMS fiber was found to afford the most efficient extraction of both volatile and semi-volatile compounds from the analyte’s headspace according to literature [34].

The HS-SPME-GC-MS analysis of raw cocoa seeds allowed the extraction of a complex mixture of VOCs and the compounds with higher intensity were selected and reported in Table 3. The key VOCs considered in this work belonged to the class of alcohols, aldehydes, esters, acids, ketones, pyrazines, and terpenes and they have all been previously reported in other works [12,34].

The main VOCs were associated with the aroma of vinegar (acetic acid) and with the aroma of roasted and nutty (tetramethyl-pyrazine). High level of acetic acid could influence in a negative way the final aroma of chocolate, moreover pyrazines were considered important contributors to the desirable chocolate aroma [12] and changed during roasting [35]. Additionally, the aldehydes 2-methylbutanal and 3-methylbutanal are reported to have a strong influence on the chocolate flavor [12]. Phenylethyl alcohol and 3-Methylbutyl acetate were considered key aroma compounds and were associated to the odor of floral, rose and sweet, fruity, banana, respectively. There were significant differences among the types of beans, in particular for the abovementioned VOCs (Table 3). The 2^nr^ (Madagascar) sample showed highest level of acetic acid and tetramethyl-pyrazine, instead of 1^nr^ (Camino Verde) showing the lowest values. O’Payo beans (sample 6^nr^) showed highest levels of the aldehydes with chocolate and almond aroma, 2-methylbutanal, 3-methylbutanal and benzaldehyde. Cheni beans (sample 5^nr^) showed highest level of banana flavor (3-Methylbutyl acetate) and Coviriali beans (sample 4^nr^) the highest value of rose aroma (Phenylethyl alcohol).

The 7^nr^ sample (Criollo) showed a large number of VOCs, not reported in Table 3, as 2-Heptanol for the class of alcohols, as 2-Pentanol acetate for esters and as 2-Heptanone for ketones. The high variety of VOCs, the low level of acetic acid and the good quantity of pyrazines and aldehydes confirmed the high quality of this variety and the possible use without roasting to produce raw chocolate.

Forastero, Criollo, Trinitario, and Nacional, the variety grown in Ecuador, exhibit differences in flavor characteristics that can be attributed to original variety but also growing conditions and geographical origin [36]. The extent to which other factors such as climate and soil chemical compositions influence the formation of flavor precursors and their relationships with final flavor quality remains unclear [36]. Some authors have studied the influence of cocoa’s origin on the composition in volatile compounds and profile comparison allowed beans, liquor, and chocolate from various geographical origins to be distinguished [29,37].

Trinitario samples from Madagascar (2^nr^) and Nicaragua (6^nr^) exhibited high differences in many of key VOCs considered and also Forastero samples from different areas of Peru, 4^nr^ and 5^nr^, showed significant differences in volatile composition.

Cocoa beans underwent the roasting process by means of a dry heat treatment, changing chocolate flavor. Flavor precursors developed during fermentation interact in the roasting process. Aldehydes and pyrazines are among the major compounds formed during roasting. They are formed through the heat induced Maillard reaction and Strecker degradation of amino acids and sugars [35]. The roasting process not only generated and increased the concentration of some flavor compounds through pyrolysis of sugars, but also reduced the amount of minor compounds affecting the final quality of chocolate [38]. The degree of chemical changes depends on the temperature applied during the process [38]. The HS-SPME-GC-MS analysis of roasted beans confirmed changes in VOCs during roasting as the decrease of acetic acid, especially in samples 4 where a higher level was present, and the increase of pyrazines in both samples (Table 3). Additionally, 3-methylbutanal increased with roasting, especially at 95 and 110 °C, while at 125 °C there was a return to initial values, due to the prevalence of the volatilization phenomenon compared to the production one. The loss of minor compounds that influence the chocolate’s aroma as phenylethyl alcohol and benzaldehyde was observed by increasing the roasting temperature, in particular at 125 °C.

### 3.3. BAs in Cocoa Beans

It can be assumed that, prior to roasting, the bacterial decarboxilation of the amino acids plays the main role in the BAs production in fresh cocoa beans. In fact, during fermentation, cocoa proteins can be hydrolyzed by microorganisms to release free amino acids, although their total amount can considerably vary [39]. Usually, low amounts of total free amino acids, mostly acidic, were detected in the unfermented seeds. It has been shown that after fermentation, acidic free amino acids decreased, while total free amino acids, as well as hydrophobic free amino acids, increased [40]. The latter aspect seems to be related to the characteristics of the aspartic endoprotease and the carboxypeptidase present in cocoa beans, as the first preferentially attacks hydrophobic amino acids of the storage proteins and the second releases single hydrophobic amino acids [40]. Considering the different optimal temperature and pH of these enzymes, proteolysis primarily depends on the fermentation conditions: duration and intensity of acidification, temperature, and aeration [39]. Once free amino acids are released, they can undergo decarboxylase activity by some bacterial enzymes to form amines [41]. Microbiota evolution during cocoa bean fermentation has been studied extensively, also owing to its importance in the formation of the precursor compounds of the cocoa flavor [42]. It was found that yeasts, filamentous fungi, lactic and acetic acid bacteria as well as members of the genus Bacillus, are typically present, all of them being able to produce BAs [43]. As a consequence of the protection mechanism of bacteria against the acid medium, decarboxylase activity is favored by low pH values during fermentation [44]. Moreover, contaminating bacteria can also decarboxylate amino acids to support a further accumulation of BAs.

In Table 4 BA distributions and total amounts in fermented, not-roasted cocoa beans of different origin are reported. Quantities of total BAs ranged from 13 mg kg^−1^ in sample 3a to 28.8 mg kg^−1^ in sample 7^nr^, never reaching hazardous concentrations. Total BAs concentrations collected in Table 4 are in agreement with a recent study, who recorded the evolution of BAs in fresh cocoa beans over a fermentation period of seven days, reaching a maximum level of 39.6 mg kg^−1^ at the fourth day of fermentation [5]. On the contrary, considering samples of the same geographical origin, Oracz and Nebesny (2014) reported for raw cocoa beans from Ecuador and Indonesia, much lower total BAs content, not exceeding 5.0 and 6.0 mg kg^−1^, respectively. However, only five BAs were considered in this study, neglecting natural polyamines PUT, SPM, and SPD, as well as, HIS and CAD, representing in our study the most abundant compounds [44].

As can be seen from data in Table 4 some variations are present, depending on the sample. This is not surprising, as it was already underlined that, when analyzing samples of cocoa beans from different countries, several attributes can be very different [45]. In fact, wide variations have been obtained considering cocoa beans coming from big producing countries, much more emphasized in samples obtained from smaller producing countries. Moreover, differences were not only country-dependent, but also farmer-dependent, as significant discrepancies were found in quality attributes of cocoa beans from the same country [39]. To this regard, it is noteworthy that samples 3^nr^ and 4^nr^ were collected from the same farm but harvested respectively in 2015 (sample 3^nr^) and 2016 (sample 4^nr^). As it can be seen, BAs profiles and concentrations did not significantly differ, implying a high degree of farming standardization.

Considering BAs profiles, the data obtained in this study clearly showed that BAs present in all samples at higher concentrations were SPM (3.5–7.2 mg kg^−1^), HIS (3.1–5.3 mg kg^−1^), PUT (1.5–3.4 mg kg^−1^), and CAD (1.1–2.1 mg kg^−1^), while TYR (not detected (nd)–4.9 mg kg^−1^), SPD (nd–7.3 mg kg^−1^) and PHE (nd–1.5 mg kg^−1^) were present more rarely and at variable concentrations. The presence of natural polyamines in the cocoa beans is expected since they are ubiquitous in plants and all living organisms. It is also known the ability of the bacteria to produce some amines, e.g., TYR, as a protection against the acidic environment, while low levels of PHE in cocoa and derivatives seem to be associated with their aphrodisiac effects and mood lifting [46]. Guillen-Casla et al. (2012) [20] reported that TYR, PHE, serotonin, and HIS were the main amines in cocoa beans, although also PUT, dopamine, and ethanolamine have also been determined. Comparison among samples of same geographical origin (samples 1 and 7), displayed comparable (Ecuador) or higher (Indonesia) amounts of PHE, while our samples always showed much higher concentration of TYR for both raw cocoa beans [44]. In addition, do Carmo Brito et al. (2017) [5] found different results. Only tryptamine, TYR, SPD, and SPM were present during fermentation of fresh cocoa beans, while CAD and PUT where always undetectable in all the analyzed samples. SPD and SPM concentrations increased from the beginning to the end of fermentation, while TYR reached its maximum level at the fourth day of fermentation, decreasing afterward to initial contents [5].

It can be concluded that the differences already recorded for total BAs concentrations are much more evident when considering BAs profiles. This is a very common situation already underlined for many other foods supporting BAs accumulation. Considering that the aminogenesis takes origin from multiple and complex variables, all of which interact, a direct overlapping of the data arising from different studies (or from different samples of the same study, if they are not produced in the same way) is generally difficult to accomplish. Many parameters concerning either the hygienic conditions of the raw materials or the production process, as well as the preservation techniques, influence BAs levels and distributions [5,16,44].

In Table 4 the evolution of BAs concentration evaluated for sample 4^nr^ and 6^nr^ at different roasting temperatures (95, 110 and 125 °C samples 4^r1^, 4^r2^, 4^r3^ and 95 and 110 °C for sample 6^r1^, 6^r2^) is reported. As can be seen, the roasting temperature is strictly related to the amine total amount, reaching for sample 6^b^ the maximum level of 58.3 mg kg^−1^, in agreement with Oracz and Nebesny (2014) [44]. They underlined that temperature and relative humidity of air during roasting influenced the BAs concentrations and profiles a lot. As can be noted from Table 4, sample 6^nr^ contained all the considered amine before roasting. Each amine concentration raised after processing, although to a different extent. In particular, PUT concentration showed the highest increasing factor (7.3) followed by TYR, CAD, HIS, PHE amounts with increasing factors between 2.1 and 2.6. SPD and SPM contents recorded the lowest enhancing, both with an increasing factor of 1.5. Although, with different profiles and distributions in comparison with Oracz and Nebesny (2014) [44], data obtained in our study confirmed the influence of the thermal processing on the increase of BAs concentrations in cocoa beans. This effect has been related to the transformation of free amino acids caused by the treatment at high temperature. In fact, it is now well established that during the Strecker degradation, the thermal decarboxylation of amino acids can occur in the presence of α-dicarbonyl compounds formed during the Maillard reaction or lipid peroxidation [19]. To this regard, literature data confirmed that asparagine, phenylalanine, and histidine changed in the corresponding amines 3-aminopropionamide, PHE, and HIS either in cocoa or in model systems [47].

As far as samples 4^nr^-^r3^ are concerned, the impact of temperature on BAs concentrations during roasting showed a different behavior. Once again, all the amines exhibited higher quantities at the end of the thermal treatment mainly HIS (increasing factor 3.3) and PUT (increasing factor 2.8), followed by CAD (increasing factor 1.6) and SPM (increasing factor 1.3). To this regard, Hidalgo et al. (2013) [47] reported that the thermal degradation of histidine was more easily produced in comparison with that of phenylalanine. This effect could explain the higher increasing factors of HIS set against with those of PHE, for both samples series 6^nr^-^r2^ and 4^nr^-^r3^.

As can be seen in Table 4, before roasting, sample 4^nr^ did not contain TYR and PHE, both appearing respectively only after a thermal treatment at 110 and 125 °C, supporting the idea that PHE is generated mainly by thermal decarboxylation of phenylalanine and not by biochemical reactions [44]. Additionally, in the case of TYR, traces of this compound, absent in green coffee (Rio quality), were detected in roasted samples after 16 min at 220 °C [48], thus demonstrating its “thermogenic” formation.

The different situations underlined by data in Table 4 probably depend on the complexity of the heat-induced formation of BAs. In fact, amines and amino acid-derived Strecker aldehydes, are simultaneously produced in food products during roasting, due to parallel pathways through the same key intermediates. Reactive carbonyl compounds started these degradations and the ratio between both aldehydes and amines generated is related to the carbonyl compound involved in the reaction and the experimental conditions, including amount of oxygen, pH, temperature, time, as well as the presence of other compounds such as antioxidants or amino acids [19]. In particular, additional amino acids were shown to play an important role in the preferential formation of either Strecker aldehydes or amino acid-derived amines by amino acid degradation in the presence of reactive carbonyl compounds. In this sense, the formation of PHE and phenylacetaldehyde in mixtures of phenylalanine, a lipid oxidation product, and a second amino acid was studied to determine the role of the second amino acid in the degradation of phenylalanine produced by lipid-derived reactive carbonyls. The presence of the second amino acid usually increased the formation of the amine and reduced the formation of the Strecker aldehyde to a differ extent depending on the considered amino acid [19]. The reasons for this behavior are not fully understood, although the obtained results suggested that they seem to be related to the other functional groups (mainly amino or similar groups) present in the side chains of the amino acid. To this regard, the limited aldehydes concentrations, especially at 125 °C (Table 3), could support this hypothesis.

Finally, the effect of antioxidants on BAs formation during roasting should be also considered. To this regard, the effect of the presence of phenolic compounds [49] on the degradation of phenylalanine, initiated by lipid-derived carbonyls was studied, to determine the structure-activity relationship of phenolics on the protection of amino compounds against modifications produced by carbonyl compounds. The obtained results showed that, among the different phenolic compounds assayed, the most efficient phenolic compounds were flavan-3-ols followed by single m-diphenols. The efficiency of these molecules was dependent on their ability to rapidly trap the carbonyl compounds. In this way the reaction of the carbonyl compound with the amino acid was avoided. This implies that the carbonyl-phenol reactions involving lipid-derived reactive carbonyls can be produced more rapidly than carbonyl-amine reactions, supporting the idea that antioxidants can provide a protection of amino compounds during thermal treatments of cocoa beans. In this sense, the loss of flavan-3-ols as the roasting temperature increased (Table 2) might be responsible of the limited BAs accumulation in the roasted cocoa beans.

## 4. Conclusions

Many classes of compounds present in cocoa nibs can be evaluated as indicators of quality and safety of raw materials and consequently of the final products.

In particular, along with a high [epicatechin]/[catechin] ratio, indicating a better bioavailability of flavanols, a high content on polyphenols could be considered as a favorable attribute of cocoa beans. This is related either to the health qualities of these compounds or to their capacity of preserving other compounds from chemical oxidation or enzymatic degradation, thus increasing stability and general characteristics of the product. According with the obtained results and taking into consideration that cocoa beans used for producing chocolate are usually roasted, the sample 6^nr^ (O’Payo 2016) appears to be the one with the best quality, showing a good content in polyphenols also after roasting at 110 °C (12.96 mg g^−1^ raw sample vs. 3.04 mg g^−1^ roasted sample. On the contrary, although showing the highest [epicatechin]:[catechin] ratio (27.9), the sample 2^nr^ seems to possess a lower quality among considered samples, in relation to its low polyphenols content (1.44 mg g^−1^).

The monitoring of the volatile aromatic fraction, as reported for the not volatile one, suggested the same conclusions. Sample 2^nr^, showed lower quality having high levels of acids that influence, in a negative way, the final aroma of chocolate. Besides, among the analyzed raw samples, low level of acetic acid and the highest levels of the aldehydes with chocolate and almond aroma confirmed the high quality of sample 6^nr^ (O’Payo 2016), as described from polyphenols analysis. The analysis of roasted beans confirmed changes in VOCs during roasting, as the decrease of acetic acid, especially in sample 6, and the increase of pyrazines associated with the nutty, cocoa, peanut-like aroma. The roasting temperature at 125 °C seemed to cause a loss of some minor compounds involved in the aroma of chocolate such as alcohols, aldehydes, and esters, resulting therefore excessive for the tested variety.

Considering BAs as cocoa quality markers as well, their total levels seem to indicate an opposite trend in comparison to that underlined by polyphenols and aroma compounds. In fact, among raw cocoa nibs, sample 6^nr^ showed the second higher BAs total concentration, indicating a medium quality among considered samples. However, it should be underlined that, after roasting at 110 °C, amine total amounts showed an increasing factor of 2.22 (6^nr^ vs. 6^r2^) and of 2.37 (4^nr^ vs. 4^r2^) implying, among the analyzed samples, a lower attitude of sample 6^nr^ to form amines during heat treatment. Anyway, from the food safety point of view, not alarming BAs amounts were found in all samples, both raw and roasted. All BAs concentrations increased after roasting, although to a different extent depending on the sample and on the considered amine. The latter aspect supports the idea that heat induced amines formation/accumulation probably during the Strecker degradation where aldehydes and amines compete to be formed, and at the same time BAs accumulation was lowered by the polyphenols intervention.

## Figures and Tables

**Table 1:** List of samples. ^nr^not roasted; ^r1^95 °C; ^r2^110 °C; ^r3^125 °C.

ID	Sample	Year	Variety	Country	Region
1^nr^	CAMINO VERDE	2015	Nacional Forastero	Ecuador	Guayas
2^nr^	MADAGASCAR	2015	Trinitario	Madagascar	Sambirano
3^nr^	COVIRIALI	2015	Forastero	Peru	Junín
4^nr^, 4^r1^, 4^r2^, 4^r3^	COVIRIALI	2016	Forastero	Peru	Junín
5 ^nr^	CHENI	2016	Forastero	Peru	Satipo
6^nr^, 6^r1^, 6^r2^	O’PAYO	2016	Trinitario	Nicaragua	North Caribbean Coast Autonomous Region
7^nr^	CRIOLLO	2016	Criollo	Indonesia	Bali

**Table 2:** HPLC/DAD quantitative analysis of methylxantines and polyphenols in cocoa nibs. Results in mg g^−1^ vegetal material. Data obtained from triplicate analysis with relative standard deviation (SD) 2–5%.

	1^nr^	2^nr^	3^nr^	5^nr^	7^nr^	4^nr^	4^r1^	4^r2^	4^r3^	6^nr^	6^r1^	6^r2^
Theobromine	8.65 ^f^	1.95^a^	7.81 ^de^	7.69 ^d^	7.61 ^d^	8.49 ^f^	8.52 ^f^	7.68 ^d^	7.14 ^c^	8.08 ^e^	10.08 ^g^	6.68 ^b^
Caffeine	2.01 ^c^	0.27^a^	1.89 ^c^	1.70 ^b^	0.96 ^b^	3.81 ^e^	2.81 ^d^	2.18 ^c^	1.60 ^b^	2.60 ^d^	2.04 ^c^	2.03 ^c^
Catechin	nd ^a^	0.01 ^b^	nd ^a^	0.17 ^g^	0.06 ^c^	0.20 ^h^	0.15 ^f^	0.10 ^d^	0.14 ^f^	0.24 ^i^	0.12 ^e^	0.12 ^e^
Epicatechin	2.84 ^i^	0.16 ^a^	0.84 ^e^	0.87 ^e^	0.32 ^b^	1.98 ^g^	1.08 ^f^	0.58 ^c^	0.73 ^d^	3.12 ^j^	2.22 ^h^	0.69 ^d^
Procyanidins	7.58 ^h^	1.00 ^b^	9.39 ^j^	8.15 ^i^	0.62 ^a^	2.47 ^f^	1.29 ^d^	1.17 ^c^	1.01 ^b^	9.17 ^j^	5.17 ^g^	1.92 ^e^
Quercetin hexoside	0.03 ^c^	0.02 ^b^	0.03 ^c^	0.05 ^d^	0.02 ^b^	<LOQ ^a^	0.07 ^e^	0.05 ^d^	0.05 ^d^	0.03 ^c^	0.05 ^d^	0.02 ^b^
Quercetin arabinoside	0.04 ^e^	0.03 ^d^	0.04 ^e^	0.05 ^f^	0.02 ^c^	<LOQ ^a^	0.09 ^h^	0.06 ^g^	0.06 ^g^	0.05 ^f^	0.10 ^i^	0.01 ^b^
Caffeoyl aspartic acid	0.12 ^b^	0.13 ^b^	0.24 ^c^	<LOQ ^a^	0.24 ^c^	0.51 ^h^	0.43 ^g^	0.76 ^i^	0.38 ^f^	0.34 ^e^	0.29 ^d^	0.28 ^d^
Hydroxycinnamic der.	0.06 ^b^	0.09 ^c^	nd ^a^	nd ^a^	nd ^a^	nd ^a^	nd ^a^	nd ^a^	nd ^a^	nd ^a^	nd ^a^	nd ^a^
N-caffeoyl-L-DOPA	<LOQ ^a^	<LOQ ^a^	<LOQ ^a^	<LOQ ^a^	<LOQ ^a^	<LOQ ^a^	<LOQ ^a^	<LOQ ^a^	<LOQ ^a^	<LOQ ^a^	<LOQ ^a^	<LOQ ^a^
Epicatechin:catechin ratio		27.9		5.0	5.0	9.7	7.0	6.0	5.3	12.9	18.7	5.9
Toal xanthines	10.66 ^d^	2.22 ^a^	9.71 ^c^	9.39 ^c^	8.58 ^b^	12.30 ^f^	11.33 ^e^	9.86 ^c^	8.74 ^b^	10.68 ^d^	12.12 ^f^	8.71 ^b^
Total polyphenols	10.67 ^i^	1.44 ^b^	10.55 ^i^	9.29 ^h^	1.28 ^a^	5.16 ^f^	3.11 ^e^	2.72 ^d^	2.37 ^c^	12.96 ^j^	7.95 ^g^	3.04 ^e^

Different letters express significant differences (*p* < 0.05). ^nr^not roasted; ^r1^95 °C; ^r2^110 °C; ^r3^125 °C. nd means not detected and <LOQ means under limit of quantification.

**Table 3:** Key aroma volatiles in raw samples and in roasted samples together with their odor descriptors, normalized peak area from Q (quantitation)-ion, and Internal Standard (IS). Data obtained from triplicate analysis with SD < 5%.

Odor Descriptor	Compound Name	1^nr^	2^nr^	4^nr^	5^nr^	6^nr^	7^nr^	4^r1^	4^r2^	4^r3^	6^r1^	6 ^r2^
	Alcohols											
Fruity, creamy, buttery	2,3-Butanediol	0.03 ^b^	0.36 ^c^	0.63 ^e^	1.31 ^f^	3.96 ^h^	nd ^a^	0.44 ^d^	0.45 ^d^	0.37 ^c^	3.22 ^g^	7.76 ^i^
floral, rose	Phenylethyl alcohol	34.98 ^e^	16.39 ^b^	72.20 ^i^	5.20 ^a^	42.72 ^f^	23.03 ^c^	77.84 ^j^	128.69 ^k^	48.64 ^h^	45.33 ^g^	27.08 ^d^
	Aldehydes											
Aldehydic, chocolate	2-methyl-Butanal	nd ^a^	nd ^a^	nd ^a^	0.003 ^b^	0.01 ^c^	0.003 ^b^	nd ^a^	nd ^a^	nd ^a^	0.01 ^c^	0.01 ^c^
aldehydic, chocolate	3-methyl-Butanal	0.02 ^b^	0.01^a^	0.02 ^b^	0.01 ^a^	0.05 ^e^	0.03 ^c^	0.03 ^c^	0.04 ^d^	0.02 ^b^	0.13 ^f^	0.13 ^f^
sweet, bitter, almond, cherry	Benzaldehyde	16.24 ^g^	4.30^b^	12.25 ^d^	1.61 ^a^	46.55 ^k^	12.98 ^e^	15.28 ^f^	26.92 ^i^	10.23 ^c^	33.36 ^j^	22.69 ^h^
	Esters											
Sweet, fruity, banana	3-Methylbutyl acetate	3.46 ^a^	15.96 ^h^	7.61 ^e^	23.13 ^j^	6.54 ^c^	17.85 ^i^	9.63 ^g^	9.38 ^f^	4.53 ^b^	7.64 ^e^	6.96 ^d^
floral, rose, honey, tropical	Acetic acid, 2-phenylethyl ester	3.38 ^b^	5.96 ^c^	22.19 ^i^	2.46 ^a^	10.54 ^d^	13.80 ^f^	17.02 ^g^	41.48 ^j^	18.03 ^h^	11.39 ^e^	10.69 ^d^
	2,3-Butanediol diacetate	0.04 ^b^	1.39 ^i^	0.44 ^f^	1.46 ^k^	0.26 ^c^	nd ^a^	0.38 ^e^	0.42 ^f^	0.74 ^h^	0.32 ^d^	0.62 ^g^
	Acids											
Acidic	2-methyl-Propanoic acid	0.17 ^e^	0.07 ^b^	0.16 ^e^	0.26 ^f^	0.38 ^i^	0.30 ^g^	0.09 ^c^	0.06 ^a^	0.15 ^d^	0.36 ^h^	0.32 ^g^
Cheese, pungent, fruity	3-methyl-Butanoic acid	0.31 ^f^	0.11 ^b^	0.22 ^d^	0.27 ^e^	0.50 ^i^	0.45 ^h^	0.13 ^c^	0.10 ^a^	0.22 ^d^	0.45 ^h^	0.40 ^g^
vinegar	Acetic acid	4.39 ^a^	14.32 ^j^	12.01 ^i^	7.85 ^f^	7.32 ^e^	7.94 ^fg^	6.18 ^c^	5.23 ^b^	8.06 ^h^	6.50 ^d^	8.01 ^g^
fatty, sweat, cheese	Hexanoic acid	nd ^a^	0.36 ^e^	0.73 ^f^	0.09 ^b^	0.26 ^c^	nd ^a^	0.36 ^e^	0.38 ^e^	0.09 ^b^	0.25 ^c^	0.30 ^d^
	4-hydroxy-Butanoic acid	0.04 ^c^	0.03 ^b^	0.06 ^d^	0.02 ^a^	0.02 ^a^	0.03 ^b^	0.02 ^a^	0.02 ^a^	0.03 ^ab^	0.02 ^a^	0.02 ^a^
	Ketones											
Sweet, pungent, caramel	2,3-Butanedione	0.02 ^a^	0.53 ^h^	0.09 ^d^	0.08 ^c^	0.17 ^g^	0.04 ^b^	0.08 ^c^	0.04 ^b^	0.08 ^cd^	0.15 ^f^	0.13 ^e^
buttery, milky, fatty	3-hydroxy-2-Butanone	0.20 ^a^	1.76 ^g^	1.40 ^e^	1.52 ^f^	2.39 ^i^	0.45 ^b^	1.38 ^e^	0.92 ^c^	1.31 ^d^	3.22 ^k^	1.91 ^h^
Sweet, pungent, mimosa, almond	Acetophenone	0.68 ^c^	0.58 ^b^	1.04 ^e^	0.08 ^a^	3.46 ^h^	0.61 ^b^	0.83 ^d^	1.01 ^e^	0.69 ^c^	2.69 ^g^	1.27 ^f^
musty, nutty, coumarin	2-acetylpyrrole	0.04 ^a^	0.40 ^d^	0.10 ^b^	0.09 ^b^	0.17 ^c^	0.53 ^e^	0.57 ^f^	1.10 ^h^	0.62 ^g^	1.58 ^h^	1.45 ^i^
	Pyrazines											
Nutty, cocoa, roasted, peanut	Trimethyl-Pyrazine	0.36 ^a^	4.63 ^i^	1.64 ^c^	1.83 ^d^	1.57 ^b^	2.29 ^e^	2.37 ^f^	2.62 ^g^	3.28 ^h^	7.67 ^k^	7.60 ^j^
Nutty, cocoa, peanut-like	Tetramethyl-Pyrazine	0.78 ^a^	76.59 ^k^	31.99 ^g^	38.88 ^h^	22.90 ^d^	18.78 ^b^	28.25 ^f^	19.54 ^c^	40.39 ^i^	58.60 ^j^	28.08 ^e^
	Terpenes											
Citrus	Limonene	0.16 ^e^	nd ^a^	0.03 ^c^	0.02 ^b^	Nd ^a^	0.05 ^d^	0.02 ^b^	0.02 ^b^	0.02 ^b^	nd ^a^	nd ^a^

Different letters express significant differences (*p* < 0.05). nd means not detected. ^nr^not roasted; ^r1^95 °C; ^r2^110 °C; ^r3^125 °C.

**Table 4:** Biogenic amines (BAs) in fermented and roasted cocoa beans samples. Results in mg kg^−1^ vegetal material.

BAs	1^nr^	2^nr^	3^nr^	4^nr^	4^r1^	4^r2^	4^r3^	5^nr^	6^nr^	6^r1^	6^r2^	7^nr^
**PHE**	1.1 ± 0.1 ^bc^	nd ^a^	nd ^a^	nd ^a^	nd ^a^	nd ^a^	1.3 ± 0.2 ^cd^	1.5 ± 0.1 ^d^	1.0 ± 0.1 ^b^	1.5 ± 0.1 ^d^	2.1 ± 0.1 ^e^	nd ^a^
**PUT**	3.4 ± 0.2 ^d^	1.5 ± 0.1 ^a^	2.5 ± 0.1 ^bc^	2.6 ± 0.1 ^c^	4.0 ± 0.1 ^e^	5.6 ± 0.2 ^g^	7.3 ± 0.2 ^h^	2.4 ± 0.1 ^b^	1.5 ± 0.1 ^a^	4.5 ± 0.2 ^f^	10.9 ± 0.3 ^i^	1.5 ± 0.1 ^a^
**CAD**	1.8 ± 0.1 ^c^	1.1 ± 0.1 ^a^	1.3 ± 0.1 ^b^	1.3 ± 0.1 ^b^	1.8 ± 0.1 ^c^	2.0 ± 0.1 ^d^	2.1 ± 0.2 ^d^	1.2 ± 0.1 ^ab^	1.1 ± 0.1 ^a^	1.9 ± 0.1 ^cd^	2.6 ± 0.2 ^f^	2.1 ± 0.1 ^e^
**HIS**	4.1 ± 0.1 ^c^	5.3 ± 0.2 ^d^	3.5 ± 0.1 ^b^	3.1 ± 0.1 ^a^	8.1 ± 0.2 ^f^	10.0 ± 0.3 ^g^	10.3 ± 0.2 ^g^	3.5 ± 0.1 ^b^	5.6 ± 0.2 ^de^	10.0 ± 0.2 ^g^	12.8 ± 0.3 ^h^	5.9 ± 0.2 ^e^
**TYR**	4.9 ± 0.2 ^d^	1.8 ± 0.1 ^b^	nd ^a^	nd ^a^	nd ^a^	6.7 ± 0.2 ^e^	7.3 ± 0.2 ^f^	4.9 ± 0.2 ^d^	4.3 ± 0.1 ^c^	10.8 ± 0.3 ^g^	11.1 ± 0.3 ^g^	4.8 ± 0.1 ^d^
**SPD**	1.3 ± 0.1 ^b^	nd ^a^	nd ^a^	nd ^a^	nd ^a^	nd ^a^	nd ^a^	1.9 ± 0.1 ^c^	6.7 ± 0.2 ^d^	9.1 ± 0.2 ^f^	9.7 ± 0.2 ^g^	7.3 ± 0.2 ^e^
**SPM**	5.8 ± 0.2 ^c^	4.5 ± 0.1 ^b^	5.9 ± 0.2 ^c^	6.0 ± 0.1 ^c^	6.5 ± 0.2 ^d^	6.5 ± 0.2 ^d^	7.3 ± 0.2 ^e^	3.5 ± 0.1 ^a^	6.1 ± 0.2 ^cd^	8.8 ± 0.2 ^f^	9.1 ± 0.2 ^f^	7.2 ± 0.2 ^e^
**Total**	22.4 ± 0.2 ^e^	14.2 ± 0.2 ^b^	13.2 ± 0.4 ^a^	12.9 ± 0.1 ^a^	20.5 ± 0.2 ^d^	30.9 ± 0.8 ^h^	35.6 ± 0.5 ^i^	18.9 ± 0. 1^c^	26.3 ± 0.2 ^f^	46.6 ± 0.7 ^j^	58.3 ± 0.3 ^k^	28.8 ± 0.4 ^g^

The values are expressed as means ± SD of three independent experiments. Different letters express significant differences (*p* < 0.05). nd means not detected or below limit of quantitation. ^nr^not roasted; ^r1^95 °C; ^r2^110 °C; ^r3^125 °C. PHE—β-phenylethylamine, PUT—putrescine, CAD—cadaverine, HIS—histamine, TYR—tyramine, SPD—spermidine, SPM—spermine.

## References

[1] Schwan R.F., Wheals A.E. (2004). The microbiology of cocoa fermentation and its role in chocolate quality. Crit. Rev. Food Sci. Nutr..

[2] Ding E.L., Hutfless S.M., Ding X., Girotra S. (2006). Chocolate prevention of cardiovascular disease: A systematic review. Nutr. Metab..

[3] Pereira-Caro G., Borges G., Nagai C., Jackson M.C., Yokota T., Crozier A., Ashihara H. (2013). Profiles of Phenolic Compounds and Purine Alkaloids during the Development of Seeds of *Theobroma cacao* cv. Trinitario. J. Agric. Food Chem..

[4] Camu N., De Winter T., Addo S.K., Takrama J.S., Bernaert H., De Vuyst L. (2008). Fermentation of cocoa beans: Influence of microbial activities and polyphenol concentrations on the flavour of chocolate. J. Sci. Food Agric..

[5] do Carmo Brito B.D.N., Campos Chisté R., da Silva Pena R., Abreu Gloria M.B., Santos Lopes A. (2017). Bioactive amines and phenolic compounds in cocoa beans are affected by fermentation. Food Chem..

[6] Hurst W.J., Krake S.H., Bergmeier S.C., Payne M.J., Miller K.B., Stuart D.A. (2011). Impact of fermentation, drying, roasting and Dutch processing on flavan-3-ol stereochemistry in cacao beans and cocoa ingredients. Chem. Cent. J..

[7] Kothe L., Zimmermann B.F., Galensa R. (2013). Temperature influences epimerization and composition of flavanol monomers, dimers and trimers during cocoa bean roasting. Food Chem..

[8] Arlorio M., Locatelli M., Travaglia F., Coïsson J.D., Del Grosso E., Minassi A., Appendino G., Martelli A. (2008). Roasting impact on the contents of clovamide (N-caffeoyl-L-DOPA) and the antioxidant activity of cocoa beans (*Theobroma cacao* L.). Food Chem..

[9] Bailey S., Mitchell D., Bazinet M., Weurman C. (1962). Studies of the volatile components of different varieties of cocoa beans. J. Food Sci..

[10] Clapperton J., Yow S., Chan J., Lim D., Lockwood R., Romanczyk L., Hammerstone J. (1994). The contribution of genotype to cocoa (*Theobroma cacao* L.) flavour. Trop. Agric..

[11] Van der Wals B., Kettenes D., Stoffelsma J., Sipma G., Semper A. (1971). New volatile components of roasted cocoa. J. Agric. Food Chem..

[12] Counet C., Callemien D., Ouwerx C., Collin S. (2002). Use of gas chromatography-olfactometry to identify key odorant compounds in dark chocolate: Comparison of samples before and after conching. J. Agric. Food Chem..

[13] Restuccia D., Spizzirri U.G., Puoci F., Picci N. (2015). Determination of biogenic amine profiles in conventional and organic cocoa-based products. Food Addit. Contam. Part A Chem. Anal. Control Expo. Risk Assess..

[14] Jairath G., Singh P.K., Dabur R.S., Rani M., Chaudhari M. (2015). Biogenic amines in meat and meat products and its public health significance: A review. J. Food Sci. Technol..

[15] Araujo Q.R.D., Gattward J.N., Almoosawi S., Parada Costa Silva M.D.G.C., Dantas P.A.D.S., Araujo Júnior Q.R.D. (2016). Cocoa and human health: From head to foot—A review. Crit. Rev. Food Sci. Nutr..

[16] Restuccia D., Spizzirri U.G., De Luca M., Parisi O.I., Picci N. (2016). Biogenic amines as quality marker in organic and fair-trade cocoa-based products. Sustainability.

[17] Bandanaa J., Egyir I.S., Asante I. (2016). Cocoa farming households in Ghana consider organic practices as climate smart and livelihoods enhancer. Agric. Food Secur..

[18] Ormanci H.B., Arik Colakoglu F. (2017). Changes in biogenic amines levels of lakerda (*Salted Atlantic Bonito*) during ripening at different temperatures. J. Food Process. Preserv..

[19] Hidalgo F.J., León M., Zamora R. (2016). Amino acid decarboxylations produced by lipid-derived reactive carbonyls in amino acid mixtures. Food Chem..

[20] Guillén-Casla V., Rosales-Conrado N., León-González M.E., Pérez-Arribas L.V., Polo-Díez L.M. (2012). Determination of serotonin and its precursors in chocolate samples by capillary liquid chromatography with mass spectrometry detection. J. Chromatogr. A.

[21] Romani A., Ieri F., Turchetti B., Mulinacci N., Vincieri F.F., Buzzini P. (2006). Analysis of condensed and hydrolysable tannins from commercial plant extracts. J. Pharm. Biomed. Anal..

[22] Sànchez-Rabaneda F., Jàuregui O., Casals I., Andrès-Lacueva C., Izquierdo-Pulido M., Lamuela-Raventòs R.M. (2003). Liquid chromatographic/electrospray ionization tandem mass spectrometric study of the phenolic composition of cocoa (*Theobroma cacao*). J. Mass Spectrom..

[23] Hammerstone J.F., Lazarus S.A., Schmitz H.H. (2000). Procyanidin Content and Variation in Some Commonly Consumed Foods. J. Nutr..

[24] Shumov L., Bodor A. (2011). An industry consensus study on an HPLC fluorescence method for the determination of (±)-catechin and (±)-epicatechin in cocoa and chocolate products. Chem. Cent. J..

[25] Spizzirri U.G., Parisi O.I., Picci N., Restuccia D. (2016). Application of LC with evaporative light scattering detector for biogenic amines determination in fair trade cocoa-based products. Food Anal. Methods.

[26] Hümmer W., Schreier P. (2008). Analysis of proanthocyanidins. Mol. Nutr. Food Res..

[27] Payne M.J., Hurst W.J., Miller K.B., Rank C., Stuart D.A. (2010). Impact of fermentation, drying, roasting, and Dutch processing on epicatechin and catechin content of cacao beans and cocoa ingredients. J. Agric. Food Chem..

[28] Ioannone F., Di Mattia C.D., De Gregorio M., Sergi M., Serafini M., Sacchetti G. (2015). Flavanols, proanthocyanidins and antioxidant activity changes during cocoa (*Theobroma cacao* L.) roasting as affected by temperature and time of processing. Food Chem..

[29] Counet C., Ouwerx C., Rosoux D., Collin S. (2004). Relationship between procyanidin and flavor contents of Cocoa liquors from different origins. J. Agric. Food Chem..

[30] Di Mattia C.D., Sacchetti G., Mastrocola D., Serafini M. (2017). From Cocoa to Chocolate: The impact of Processing on In Vitro Antioxidant Activity and the effects of Chocolate on Antioxidant Markers In Vivo. Front. Immunol..

[31] Oracz J., Zyzelewicz D., Nebesny E. (2015). The content of polyphenolic compounds in cocoa beans (*Theobroma cacao* L.), depending on variety, growing region and processing operations: A review. Crit. Rev. Food Sci. Nutr..

[32] Tomas-Barberaän F.A., Cienfuegos-Jovellanos E., Marìn A., Muguerza B., Gil-Izquierdo A., Cerdà B., Zafrilla P., Morillas J., Mulero J., Ibarra A. (2007). A New Process to Develop a Cocoa Powder with Higher Flavonoid Monomer Content and Enhanced Bioavailability in Healthy Humans. J. Agric. Food Chem..

[33] Afoakwa E.O., Quao J., Takrama J., Simpson Budu A., Saalia F.K. (2013). Chemical composition and physical quality characteristics of Ghanaian cocoa beans as affected by pulp pre-conditioning and fermentation. J. Food Sci. Technol..

[34] Ducki S., Miralles-Garcia J., Zumbé A., Tornero A., Storey D.M. (2008). Evaluation of solid-phase micro-extraction coupled to gas chromatography–mass spectrometry for the headspace analysis of volatile compounds in cocoa products. Talanta.

[35] Heinzler M., Eichner K. (1992). The role of amodori compounds during cocoa processing—Formation of aroma compounds under roasting conditions. Z. Lebensm.-Unters.-Forsch..

[36] Kongor J.E., Hinneha M., Van deWalle D., Ohene Afoakwa E., Boeckx P., Dewettinck K. (2016). Factors influencing quality variation in cocoa (*Theobroma cacao*) bean flavour profile—A review. Food Res. Int..

[37] Cambrai A., Marcic C., Morville S., Sae Houer P., Bindler F., Marchioni E. (2010). Differentiation of chocolates according to the Cocoa’s geographical origin using chemometrics. J. Agric. Food Chem..

[38] Ramli N., Hassan O., Said M., Samsudin W., Idris N.A. (2006). Influence of roasting conditions on volatile flavor of roasted malaysian cocoa beans. J. Food Process. Preserv..

[39] Rohsius C., Matissek R., Lieberei R. (2006). Free amino acid amounts in raw cocoas from different origins. Eur. Food Res. Technol..

[40] Adeyeye E.I., Akinyeye R.O., Ogunlade I., Olaofe O., Boluwade J.O. (2010). Effect of farm and industrial processing on the amino acid profile of cocoa beans. Food Chem..

[41] Granvogl M., Bugan S., Schieberle P. (2006). Formation of amines and aldehydes from parent amino acids during thermal processing of cocoa and model systems: New insights into pathways of the Strecker reaction. J. Agric. Food Chem..

[42] Lima L.J.R., Almeida M.H., Nout M.J.R., Zwietering M.H. (2011). Theobroma cacao L., “the food of the gods”: Quality determinants of commercial cocoa beans, with particular reference to the impact of the fermentation. Crit. Rev. Food Sci. Nutr..

[43] Schwan R.F., Pereira G.V.M., Fleet G.H., Schwan R.F., Fleet G.H. (2014). Microbial activities during cocoa fermentation. Cocoa and Coffee Fermentations.

[44] Oracz J., Nebesny E. (2014). Influence of roasting conditions on the biogenic amine content in cocoa beans of different *Theobroma cacao* cultivars. Food Res. Int..

[45] Caligiani A., Cirlini M., Palla G., Ravaglia R., Arlorio M. (2007). GC/MS detection of chiral markers in cocoa beans of different quality and geographic origin. Chirality.

[46] Shukla S., Park H.K., Kim J.K., Kim M. (2010). Determination of biogenic amines in Korean traditional fermented soybean paste (Doenjang). Food Chem. Toxicol..

[47] Hidalgo F.J., Navarro J.L., Delgado R.M., Zamora R. (2013). Histamine formation by lipid oxidation products. Food Res. Int..

[48] Oliveira S.D., Franca A.S., Gloria M.B.A., Borges M.L.A. (2005). The effect of roasting on the presence of bioactive amines in coffees of different qualities. Food Chem..

[49] Hidalgo F.J., Delgado R.M., Zamora R. (2017). Protective effect of phenolic compounds on carbonyl-amine reactions produced by lipid-derived reactive carbonyls. Food Chem..

